# Influence of water temperature and food on the last stages of cultured pearl mineralization from the black-lip pearl oyster *Pinctada margaritifera*

**DOI:** 10.1371/journal.pone.0193863

**Published:** 2018-03-05

**Authors:** Oïhana Latchere, Vincent Mehn, Nabila Gaertner-Mazouni, Gilles Le Moullac, Julie Fievet, Corinne Belliard, Philippe Cabral, Denis Saulnier

**Affiliations:** 1 Ifremer, UMR 241 « Ecosystèmes Insulaires Océaniens », Labex Corail, Centre du Pacifique, Tahiti, French Polynesia; 2 Université de la Polynésie Française, UMR 241 « Ecosystèmes Insulaires Océaniens », Labex Corail, Faa’a, Tahiti, French Polynesia; 3 Gauguin’s Pearl Farm, Rangiroa, French Polynesia; Laboratoire de Biologie du Développement de Villefranche-sur-Mer, FRANCE

## Abstract

Environmental parameters, such as food level and water temperature, have been shown to be major factors influencing pearl oyster shell growth and molecular mechanisms involved in this biomineralization process. The present study investigates the effect of food level (i.e., microalgal concentration) and water temperature, in laboratory controlled conditions, on the last stages of pearl mineralization in order to assess their impact on pearl quality. To this end, grafted pearl oysters were fed at different levels of food and subjected to different water temperatures one month prior to harvest to evaluate the effect of these factors on 1) pearl and shell deposition rate, 2) expression of genes involved in biomineralization in pearl sacs, 3) nacre ultrastructure (tablet thickness and number of tablets deposited per day) and 4) pearl quality traits. Our results revealed that high water temperature stimulates both shell and pearl deposition rates. However, low water temperature led to thinner nacre tablets, a lower number of tablets deposited per day and impacted pearl quality with better luster and fewer defects. Conversely, the two tested food level had no significant effects on shell and pearl growth, pearl nacre ultrastructure or pearl quality. However, one gene, Aspein, was significantly downregulated in high food levels. These results will be helpful for the pearl industry. A wise strategy to increase pearl quality would be to rear pearl oysters at a high water temperature to increase pearl growth and consequently pearl size; and to harvest pearls after a period of low water temperature to enhance luster and to reduce the number of defects.

## Introduction

As in other mollusks, pearl oysters synthesize biomineralized structures, such as their shell, to maintain their soft tissues, and to prevent predation and desiccation [1]. Shell biomineralization results from the activity of an organic matrix, mostly composed of polysaccharides, lipids and proteins, secreted by the mantle tissue. The shell is composed of different layers: the periostracum, a thin layer mainly consisting of organic material; the outer prismatic layer made of calcite; and the inner nacreous layer made of aragonite. The nacreous layer has an iridescent and shiny appearance and is of great interest for cultured pearl production. To produce a cultured pearl, a small piece of mantle is cut from a donor oyster and is then implanted together with a nucleus (consisting of mollusk shell or synthetic material) into the gonad of a recipient oyster [2]. The epithelial cells of the graft multiply, ending in the formation of a complete pearl sac covering the nucleus. Histological examinations in *P*. *margaritifera* revealed that the pearl sac is complete after approximately 14 days following the graft operation [2]. At 18 days post grafting, the pearl-sac cannot be distinguished from the host tissues [2]. As early as 21 days post grafting, the nucleus is partially or totally covered by the first secretions, made of both organic and mineral materials, due to the mineralizing activity of the pearl sac [3]. In this study, huge diversity in the microstructural patterns and mineralogical properties was observed in the first pearl layers of one-month or older pearls, until a homogeneous nacreous layer occurred in most pearls. Another study focusing on the chronological description of pearl-sac development showed that first nacre deposition was recorded at 32 days post grafting [2]. The nacreous layers are composed of aragonite tiles held together by a series of organic matrices [4]. The laminar structure and the thickness of nacre piled on the implanted nucleus are considered as determinant factors for pearl quality [5]. Interestingly, the gene expression patterns of shell matrix proteins (SMP) in pearl sacs are very similar to that of the donor mantle tissue [6]. Shell matrix proteins are known to control shell biomineralization by determining the type of calcium carbonate (calcite or aragonite) that will be deposited and by regulating crystal growth [7]. A large research effort has been conducted to identify and characterize mineralization-related proteins and genes [7–11]. For example, Pif177, MSI60 and Pearlin have been identified as being involved in the formation of a nacreous layer, respectively by specifically binding to aragonite crystals [12], by including a calcium-binding domain [13] and by presenting calcium- and chitin-binding properties that would be involved in nacre crystal structures development [14]. Furthermore, Aspein is related to the prismatic layer and contains an aspartic-rich domain which might be involved in controlling selective precipitation of calcite [15–17]. Other proteins are involved in both the formation of nacreous and prismatic layers, such as Nacrein, which is thought to act as a calcium concentrator [18].

Pearl production in French Polynesia is an important industry, with production sites located in the Society, Gambier and Tuamotu archipelagos. Tahitian cultured pearls produced from the pearl oyster *Pinctada margaritifera* are harvested after approximately 18 to 24 months. Their quality is evaluated using different criteria: size, surface quality, luster, shape, color and darkness level [19]. Sizes range from 9 to 20 mm; with average size around 8–12 mm [20]. Surface quality is an assessment of the different imperfections on the surface of the pearl, such as spots, bumps and wrinkles. Luster is considered as the brilliance of the pearl, where high luster corresponds to mirror-like appearance and low luster corresponds to dull appearance. The most valuable pearls are the lustrous, large, round pearls with no defects on the surface. The darker a Tahitian pearl is, the more valuable it will usually be [21]. However, only 5–10% of cultured pearls are considered to be of high quality [22]. The potential for pearl quality improvement is thus considerable.

Environmental parameters, such as food level and water temperature, have been shown to be major factors influencing pearl oyster shell growth and molecular mechanisms involved in this biomineralization process [23]. It has also been reported that food level influences the thickness of nacre tablets in the shell [24] and that pearl oyster metabolism is dependent on water temperature [25]. As a recipient oyster is strongly suspected to regulate pearl sac metabolism [26], food level and water temperature may influence pearl biomineralization. However, to our knowledge, few publications have addressed the issue of environmental influence on pearl biomineralization. Water temperature is suspected to be determinant for obtaining high-quality pearls; therefore, pearls are preferentially harvested in winter [27,28]. Indeed, lower water temperatures are assumed to reduce pearl oyster metabolism and to influence nacreous structure in the last layers of the pearl, with thinner nacre tablets that would result in higher luster [28,29]. Recently, pearl nacre growth and tablet thickness have been shown to be influenced by water temperature [30]. Finally, the iridescence and color of the shell depend on the last few layers of nacre and on the structure of nacre [31]. Therefore, the last stages of pearl mineralization may be crucial to obtaining high-quality pearls, regarding surface quality, luster and iridescence.

The present study investigates the effect of food level and water temperature, in laboratory controlled conditions, on the last stages of pearl mineralization and on pearl quality. To this end, grafted pearl oysters *P*. *margaritifera* were fed at different food levels and subjected to different water temperatures during the preharvest phase. We studied the influence of these environmental parameters on biomineralization-related gene expression in the pearl sac, and on pearl and shell growth, pearl nacre tablet thickness and pearl quality traits.

## Material and methods

### Biological material

Cultivated pearl oysters *P*. *margaritifera* of approximately two years old were reared in Rangiroa atoll (Tuamotu Archipelago, French Polynesia). A first graft was conducted in March 2014 and a second graft in May 2014. For each graft experiment, donor oyster selection was based on shell appearance and muscle resistance prior to shell opening [32]. Each recipient oyster was grafted using a 2.4 BU “Bio-coat” nucleus (7.27 mm diameter, Hyakusyo, Japan). A single professional grafter performed the two experimental grafts.

Grafted pearl oysters were then placed individually in net retention bags, and at 45 days post-grafting, nucleus retention rates (absence of rejected pearl in the retention bag) were evaluated. The pearl oysters that had retained their nucleus were hung on labeled chaplets (ropes) [22,33]. For each of the two experimental grafts, pearl oysters were transferred by plane to Ifremer Center in Vairao lagoon (Society Archipelago, French Polynesia) 9.5 months postgrafting. Grafted pearl oysters stayed in Vairao lagoon for six weeks before the experiments were conducted 11 months postgrafting.

### Experimental design

#### Experiment 1: Microalgae concentration experiment

A total of 210 pearl oysters of a mean height of 12.1 ± 1.1 cm (dorso-ventral axis) and mean weight of 257.0 ± 57.3 g were cultured in eight 500 L tanks (3 tanks with 26 pearl oysters and 1 tank with 27 pearl oysters for each condition). Pearls oysters were divided into two groups for which microalgal concentrations were gradually increased over a period of 5 days before the beginning of the experiment. Pearl oysters were then fed a mixed diet composed of two microalgae: 2/3 *Tisochrysis lutea* (T-iso) and 1/3 *Chaetoceros gracilis* at a concentration of 7,000 cell mL^-1^ (n = 105 individuals) or 28,000 cell mL^-1^ (n = 105 individuals) supplied continuously using Blackstone dosing pumps (Hanna) during one month. These two food levels are contrasted in terms of ingestion, with the higher food level being close to the maximal assimilation efficiency for *P*. *margaritifera* [34]. Homogenization of the water was achieved in the tanks by “air-lifts,” and photoperiod was maintained at 12:12. Seawater was renewed at a rate of 96L.h^-1^. Tanks were cleaned two times per week and they were sampled automatically every 3 min for fluorescence and water temperature measurements. During this experiment, the mean water temperature was 28.5 ± 0.5°C.

#### Experiment 2: Water temperature experiment

A total of 116 pearl oysters of a mean height of 12.0 ± 1.2 cm (dorso-ventral axis) and mean weight of 256.0 ± 64.3 g were cultured in eight 500 L tanks. Pearl oysters were divided into two groups for which water temperatures were gradually increased or decreased over a period of 5 days before the beginning of the experiment. Pearl oysters were then exposed to a water temperature of 22°C (n = 56 pearl oysters, divided into 14 pearl oysters per tank) or 30°C (n = 60 pearl oysters, divided into 15 pearl oysters per tank) for one month. The pearl oysters were fed a mixed diet composed of two microalgae: 2/3 *Tisochrysis lutea* (T-iso) and 1/3 *Chaetoceros gracilis* at a concentration of 15,000 cell mL^-1^ supplied continuously using Blackstone dosing pumps (Hanna). Homogenization of the water was achieved in the tanks by “air-lifts,” and photoperiod was maintained at 12:12. Seawater was renewed at a rate of 96L.h^-1^. Tanks were cleaned two times per week and they were sampled automatically every 3 min for fluorescence and water temperature measurements. In French Polynesia, the water temperature rarely drops below 22°C or exceeds 30°C. The lower water temperature is recorded in the Gambier and Austral archipelagoes, whereas the higher water temperature is recorded in the Tuamotu archipelago.

### Sampling

In order to avoid a donor oyster effect on pearl biomineralization [6,32,35], care was taken to split grafts from each donor oyster across the two treatments for each experiment (food and water temperature). From 20 grafted pearl oysters, with five donors per condition, a total of 40 pearl sacs were kept in RNAlater^®^ for each experiment (food and water temperature). A random selection of nine recipient oysters per condition and per experiment, that were implanted with grafts collected from three of the previously mentioned five donor oysters, were analyzed for pearl growth, pearl nacre tablet thickness and number of tablets deposited per day.

### Pearl sac gene expression

Total cellular RNA was extracted from the pearl sacs of *P*. *margaritifera* using TRIzol reagent (Life Technologies) according to the manufacturer’s recommendations. RNA was quantified using a NanoDrop ND-1000 spectrophotometer (NanoDrop Technologies Inc.). For each sample, 3 μg of total RNA was treated with DNase (Ambion) to degrade any potential DNA contaminants. The expression levels of five biomineralization-related genes were analyzed using quantitative reverse transcription-polymerase chain reaction (RT-qPCR) analysis using a set of forward and reverse primers (Table 1). Three other genes were used as housekeeping genes: 18S rRNA [36], glyceraldehyde 3-phosphate dehydrogenase or GAPDH [37], and an export factor binding protein or REF1 [23]. First-strand cDNA was synthesized from 500 ng of total RNA using a Transcriptor First Strand cDNA Synthesis Kit (Roche) and a combination of random hexamer and oligo(dT)primers in a final reaction volume of 25 μL. Quantitative PCR (qPCR) amplifications were carried out on a Stratagene MX3000P using Brilliant II SYBR Green QPCR Master Mix (Stratagene) with 400 nM of each primer and 10 μL of 1:100 diluted cDNA template.

**Table 1:** Set of forward and reverse primers used for gene expression analysis.

Gene	GenBank Accession Numbers	Forward primer (5′-3′)	Reverse primer (5′-3′)
**Pmarg-MSI60**	SRX022139*	TCAAGAGCAATGGTGCTAGG	GCAGAGCCCTTCAATAGACC
**Pmarg-PIF 177**	HE610401	AGATTGAGGGCATAGCATGG	TGAGGCCGACTTTCTTGG
**Pmarg-Pearlin**	DQ665305	TACCGGCTGTGTTGCTACTG	CACAGGGTGTAATATCTGGAACC
**Pmarg-Nacrein A1**	HQ654770	CTCCATGCACAGACATGACC	GCCAGTAATACGGACCTTGG
**Pmarg-Aspein**	SRX022139*	TGAAGGGGATAGCCATTCTTC	ACTCGGTTCGGAAACAACTG

*SRA accession number; EST library published in Joubert et al., 2011.

The qPCR reactions consisted of an initial step of 10 min at 95°C followed by 40 cycles (95°C for 30 s, 60°C for 30 s, and 72°C for 1 min). At the end of these steps, an additional cycle was performed from 55 to 95°C, increasing by 0.1°C every second, to generate dissociation curves and verify the specificity of the PCR products. All measurements were performed on duplicate samples. Expression levels were estimated by evaluating the fluorescence signal emitted by SYBR-Green^®^. This fluorescent marker binds to double-stranded DNA (dsDNA) and the fluorescence emitted is proportional to the dsDNA present in the reaction mix. Calculations were based on cycle threshold (Ct) values. The relative gene expression ratio of each biomineralization-related gene was calculated following the delta-delta method normalized to three reference genes [38]. This is defined in the following equation: ratio = 2^-[ΔCt sample-ΔCt calibrator]^ = 2^-ΔΔCt^, where ΔCt sample is the ΔCt obtained for a target gene in one sample after normalization to the reference genes, and ΔCt calibrator is the mean of the ΔCt values obtained for all five genes as in [23,39,40].

### Shell and pearl labeling and deposition rate

**Fig 1:**
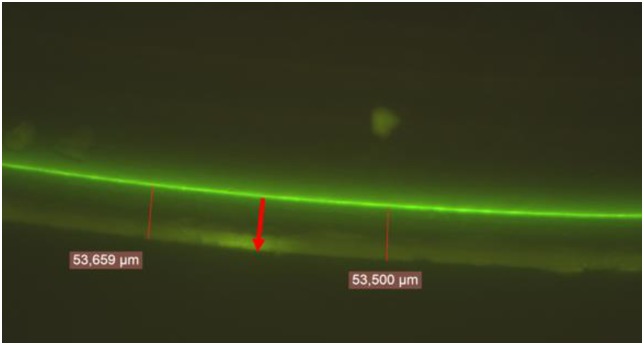
Picture of calcein marking (green fluorescent line) on a pearl cut in half and observed under an epifluorescence microscope (×400). The red arrow indicates the growth direction of nacre.

The pearl oysters were immersed for 12 h in a 150 mg.L^-1^ calcein (Sigma Aldrich) solution prepared with 0.1 mm filtered seawater. Due to technical constraint, all the pearl oysters could not be labeled at one time. Pearl oysters were divided into 4 groups that were labeled successively during 2 days. The pearl oysters then stayed in the lagoon for at least five days. Then 300 μL of calcein solution (200 mg.L^-1^, 0.1 mm filtered and sterilized seawater) was injected in the gonad of the pearl oysters using a sterile syringe. The pearl oysters stayed in the lagoon for five additional days before being transferred to the tanks. Microalgal concentrations and water temperatures were gradually increased or decreased over a period of 5 days before the beginning of the experiments. Once the microalgal concentrations and water temperatures were set, pearl oysters were fed at different microalgal concentrations (experiment 1) or subjected to different water temperatures (experiment 2) during one month. After the one-month experimental period, all shells and a selection of pearls were sawn along the dorsoventral axis and in half, respectively, using a “SwapTop Trim Saw” machine (Inland, Middlesex, United Kingdom). The ventral sides of shell cross sections and pearl sections were observed by epifluorescence microscopy using a Leica DM400B UV microscope (I3 filter block and LAS V.8.0 software for size measurements) (Fig 1). The shell and pearl deposition rates (SDR and PDR), expressed in μm day^-1^, were calculated by dividing the thickness of the new nacre deposits formed by the number of days elapsed since the marking [24].

### Pearl nacre tablet thickness and number of tablets deposited per day

Fractures of pearl were observed at 15 kV (in charge-up reduction mode) using a Hitachi TM 3030 scanning electron microscope (SEM) at the Université de la Polynésie française. High magnification images (10,000x) of fractured pearl were taken to measure the thickness of nacre tablets. For each pearl, the mean of nacre tablets thickness was calculated based on 30 measurements among the last deposited nacre layers on three different pictures (10 measurements/picture). The number of tablets deposited per day was calculated by individually dividing the pearl daily deposition rate by the mean tablet thickness.

### Evaluation of pearl quality

Cultured pearls were cleaned by ultrasonication in soapy water using an LEO 801 laboratory cleaner (2 L capacity, 80 W, 46 kHz) and were then rinsed in water. Four cultured pearl quality traits were determined: surface defects, color, darkness and luster. Surface defects were classified into one of three categories: 0 to 5 defects, 6 to 10 defects and up to 10 defects. Some pearls develop pearl surface anomaly corresponding to the presence of circle. Such circle was classified as a defect. The visually-perceived color was classified into seven color categories: grey, white, yellow, green, aubergine (red/purple), blue, and peacock (a mix of aubergine and green); and into three darkness categories: low, moderate and high. Luster was classified into three categories: low (matte appearance), moderate and high (mirror-like reflectivity). These quality traits were evaluated visually by two operators who examined the pearls together.

### Statistical analysis

The normality of data distribution and homogeneity of variance were tested using the Shapiro-Wilk and Bartlett tests, respectively. The SDR, PDR and number of tablets deposited per day followed the conditions for application of parametric tests for both experiments, so the effect of microalgal concentration and water temperature on these parameters was tested using a Student’s *t*-test. The nacre tablet thickness in pearls did not meet the conditions of application for parametric tests and could not be normalized by mathematical transformation; therefore, the effect of microalgal concentration and water temperature on nacre tablet thickness was tested using a Mann-Whitney test. The effects of the two environmental parameters on pearl surface defect classes, color and luster was tested using F-tests, whereas their effect on darkness level was evaluated using a Chi-square test. Expression values of genes that met the conditions for parametric tests were analyzed using Student’s *t*-tests, while those that did not were tested using Mann-Whitney tests. Spearman’s correlation coefficients were calculated to measure the strength of the relationships between gene expression in pearl sacs and both PDR and nacre tablet thickness, as well as between the PDR and nacre tablet thickness, at the 5% alpha level. A Pearson’s correlation coefficient was calculated to measure the linear correlation between the SDR and the PDR at the 5% alpha level with critical value r = 0.468. In all cases, p values ≤ 0.05 were considered statistically significant, and all data analyses were performed using XLSTAT (version 1.01, 2014).

## Results

### Gene expression in the pearl sac

**Fig 2:**
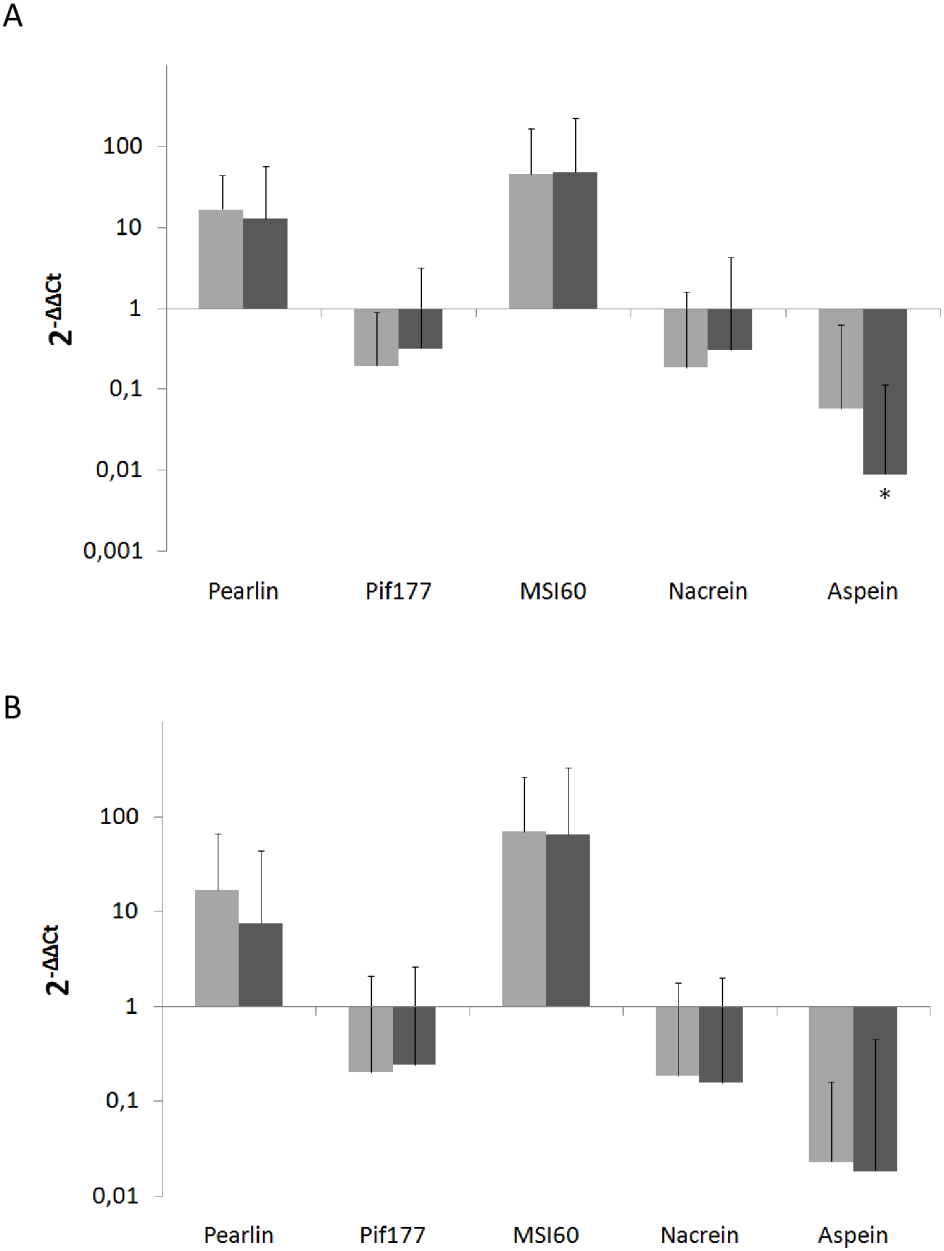
Mean relative expression of genes encoding proteins involved in the formation of the nacreous layer (Pearlin, Pif177 and MSI60), prismatic layer (Aspein) and both (Nacrein) in pearl sacs following a one-month experiment at different microalgal or water temperature conditions. (A) Different microalgal concentrations: 7,000 cell mL^-1^ (light grey) and 28,000 cell mL^-1^ (dark grey); and (B) different water temperatures: 22°C (light grey) and 30°C (dark grey). Fold changes were calculated from 20 individuals per condition. Y-axes are in the logarithmic scale. Error bars indicate standard deviations (SD); statistical analyses used Student’s *t*-tests (A: Pearlin, Pif177, MSI60 and Aspein; B: Pearlin, Pif177 and Nacrein) or Mann-Whitney tests (A: Nacrein; B: MSI60 and Aspein). Statistical significance is indicated by asterisks as follows: * p < 0.05.

Among the studied genes, only one, Aspein, was significantly affected by food level with a down-regulation for pearl oysters fed at 28,000 cell mL^-1^ compared to 7000 cell mL^-1^ (Fig 2A). Concerning the water temperature experiment, the relative expressions of the five genes were not significantly different between the two tested water temperatures (Fig 2B).

### Pearl and shell deposition rate

**Fig 3:**
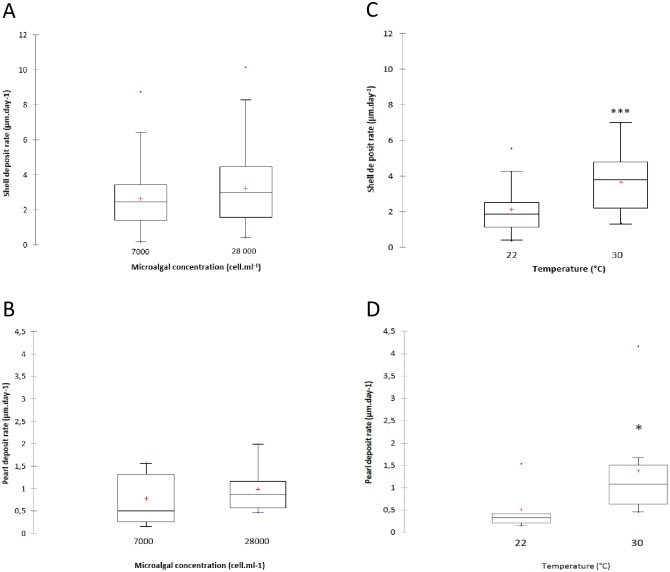
Mean shell and pearl deposition rate (SDR and PDR, μm day^-1^) in the pearl oyster *Pinctada margaritifera* after one month at different (A and B) microalgal concentrations and (C and D) water temperatures. Each plot includes mean (“+” cross in the box-plot), median (solid bar in the box-plot), 25th to 75th percentile represented in the rectangular box, 1.5×interquartile range (non-outlier range of the box whiskers), minimum and maximum values (extreme dots) and outlier values (outside box whiskers). Statistical analysis is based on the Student’s *t*-test. Statistical significance is indicated by asterisks as follows: *p < 0.05, **p < 0.01. (A: n = 88 and n = 87 for pearl oysters fed at 7000 cell mL^-1^ and 28,000 cell mL^-1^, respectively; C: n = 46 and n = 50 for pearl oysters subjected to 22°C and 30°C, respectively; and D: n = 9 per condition).

Mean shell deposition rate (SDR) and pearl deposition rate (PDR) were measured following calcein marking to analyze the effect of microalgal concentration and water temperature on shell and pearl growth during the last stages of pearl formation. Recipient oysters fed at 7000 cell mL^-1^ and 28,000 cell mL^-1^ had a mean SDR of 2.63 ± 1.6 μm day^-1^ and 3.22 ± 2.1 μm day^-1^, respectively (Fig 3A). Concerning the pearl, pearls oysters fed at 7000 cell mL^-1^ and 28,000 cell mL^-1^ produced a PDR of 0.78 ± 0.58 μm day^-1^ and 0.98 ± 0.51 μm day^-1^, respectively (Fig 3B). Student’s *t*-tests did not reveal significant differences in the SDR (p = 0.069) and in the PDR (p = 0.449) between the two microalgal concentrations.

The mean SDR was significantly lower for pearl oysters maintained at 22°C compared with those maintained at 30°C, with values of 2.13 ± 1.29 μm day^-1^ and 3.76 ± 1.88 μm day^-1^, respectively (Student’s *t*-test, p < 0.0001) (Fig 3C). Recipient oysters subjected to 22 and 30°C had PDR values of 0.51 ± 0.4 μm day^-1^ and 1.38 ± 1.1 μm day^-1^ respectively (Fig 3D). A Student’s *t*-test confirmed the significant effect of water temperature on PDR (p = 0.01).

For both experiments, SDR and PDR were strongly correlated (r = 0.668, p = 0.003 for the microalgae concentration experiment, and r = 0.659, p = 0.003 for the water temperature experiment. The critical value of Pearson’s r for both experiments was 0.468).

### Pearl nacre tablet thickness and number of tablets deposited per day

**Fig 4:**
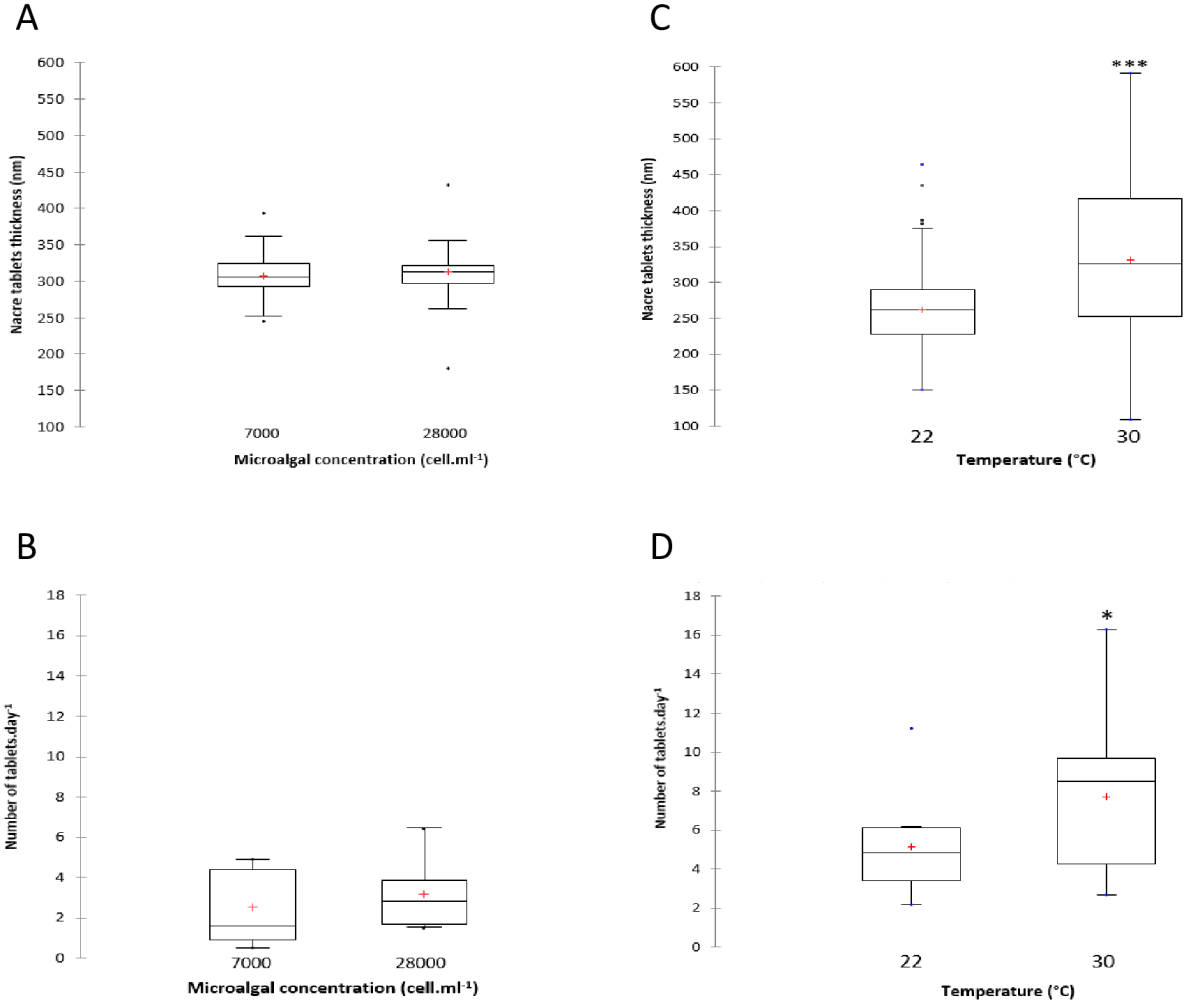
Pearl nacre tablet thickness and number of tablets deposited per day in pearl oyster *Pinctada margaritifera* after one month at different (A and B) microalgal concentrations and (C and D) water temperatures. Each plot includes: mean (“+” cross in the box-plot), median (solid bar in the box-plot), 25th to 75th percentiles represented in the rectangular box, 1.5×interquartile range (non-outlier range of the box whiskers), minimum and maximum values (extreme dots) and outlier values (outside box whiskers). Statistical analysis is based on the Mann-Whitney test for pearl nacre tablet thickness and on the Student’s *t*-test for number of tablets deposited per day. Statistical significance is indicated by asterisks as follows: *p < 0.05, ***p < 0.001. (A and C: n = 270 per condition; B and D: n = 9 per condition).

Recipient oysters fed at 7000 and 28,000 cell mL^-1^ produced pearls with mean nacre tablet thickness of 307.6 ± 11.8 nm and 312.9 ± 11.2 nm, respectively (Fig 4A). The microalgal concentrations supplied to recipient oysters showed no significant effects on nacre tablet thickness in pearls (Mann-Whitney test, p = 0.121). The pearl oysters fed at 7000 and 28,000 cell mL^-1^ exhibited a mean number of tablets deposited per day of 2.53 ± 1.87 and 3.16 ± 1.68, respectively. The microalgal concentration showed no significant effects on the mean number of tablets deposited per day in the pearls (Student’s *t*-test, p = 0.461).

The mean nacre tablet thickness was significantly lower for pearl oysters maintained at 22°C than the pearl oysters maintained at 30°C, with values of 262 ± 36.7 nm and 331.1 ± 99.4 nm, respectively (Mann-Whitney test, p = 0.042) (Fig 4C). Recipient oysters subjected to 22°C and 30°C exhibited a mean number of tablets deposited per day of 1.98 ± 1.84 and 4.05 ± 2.46 (Fig 4D). A Student’s *t*-test confirmed the significant effect of water temperature on the number of tablets deposited per day in the pearls (p = 0.023).

Among the tested genes, Pmarg-MSI60 was found to be strongly positively correlated with the PDR (ρ = 0.507, p = 0.033) and Pmarg-Aspein was negatively correlated with the PDR (ρ = -0.668, p = 0.003) for the microalgal concentration experiment (Table 2). The PDR and the tablet thickness were not significantly correlated (ρ = 0.055, p = 0.827). Concerning the water temperature experiment, we found no significant correlations between the gene expression levels and the PDR or nacre tablet thickness (Table 3). The PDR and nacre tablet thickness were not significantly correlated (ρ = 0.346, p = 0.159).

**Table 2:** Correlation between relative gene expression and pearl deposition rate and nacre tablet thickness for microalgal concentration experiment.

Gene	PDR		Nacre tablet thickness	
	ρ	p-value	ρ	p-value
Pmarg-Pearlin	0.282	0.255	-0.245	0.327
Pmarg-Pif177	0.212	0.395	-0.212	0.398
Pmarg-MSI60	**0.507**	**0.033**	-0.352	0.152
Pmarg-Nacrein A1	0.251	0.312	-0.418	0.827
Pmarg-Aspein	**-0.668**	**0.003**	-0.063	0.446

**Table 3:** Correlation between relative gene expression and pearl deposition rate and nacre tablet thickness for water temperature experiment.

Gene	PDR		Nacre tablet thickness	
	ρ	p-value	ρ	p-value
Pmarg-Pearlin	-0.195	0.436	-0.104	0.680
Pmarg-Pif177	0.067	0.789	0.059	0.815
Pmarg-MSI60	0.071	0.776	0.232	0.350
Pmarg-Nacrein A1	-0.212	0.398	0.247	0.320
Pmarg-Aspein	0.079	0.751	-0.117	0.664

### Pearl quality traits

**Fig 5:**
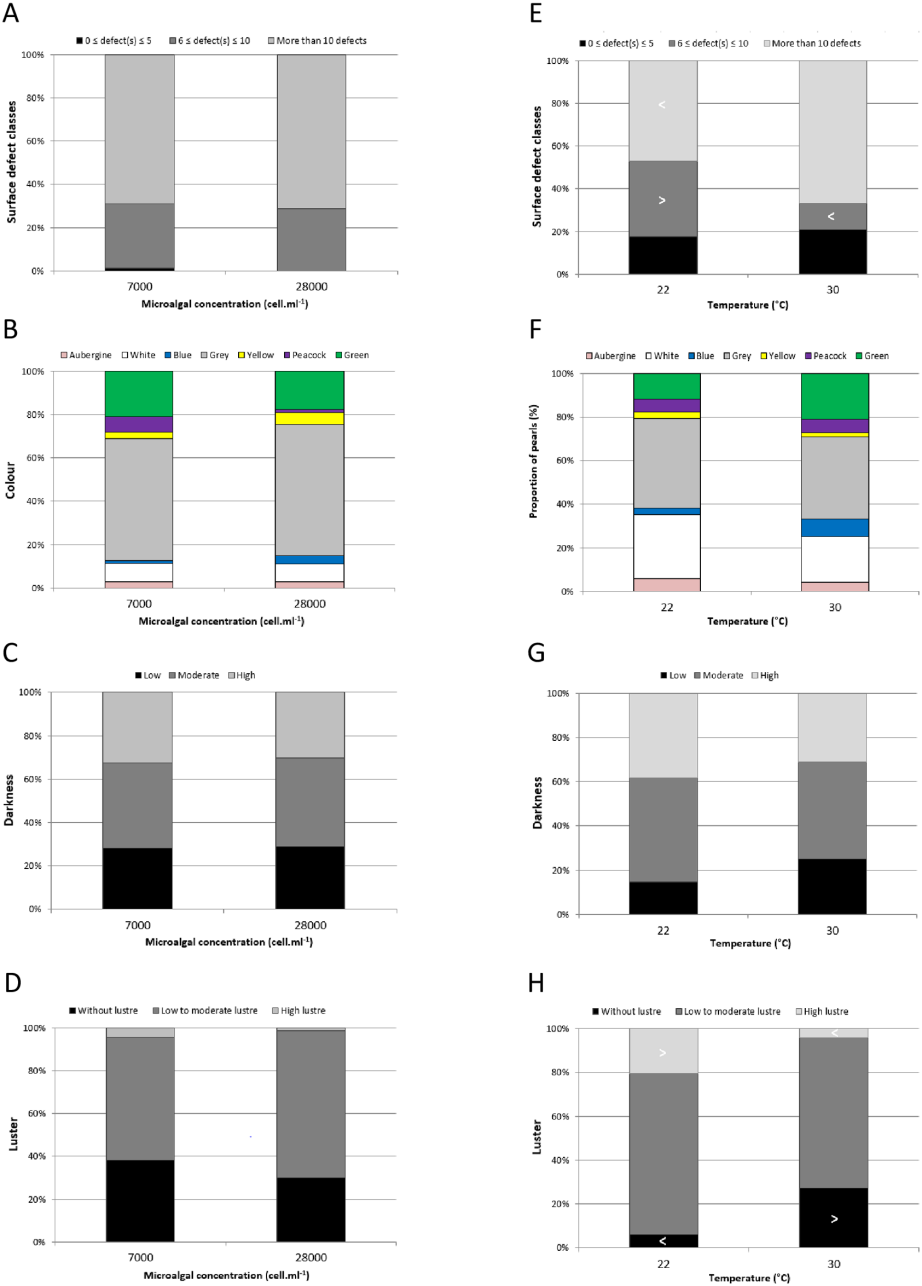
Proportion of harvested pearls in different pearl quality categories 12 months postgrafting following one month at different microalgal concentrations and water temperatures. Effect of pearl oyster fed at 7,000 or 28,000 cells mL^-1^ (n = 71 and n = 73, respectively) on (A) the number of defects, (B) color, (C) darkness and (D) luster. Effect of pearl oyster subjected to 22 or 30°C (n = 34 and n = 48, respectively) on (E) the number of defects, (F) color, (G) darkness and (H) luster. > indicates a significantly higher number and < a significantly lower number than expected by chance (F-test, p < 0.05).

The quality of pearls harvested at 12 months postgrafting was evaluated using four criteria: surface quality as the number of defects, visual color and darkness level, and luster (S1 Data and Fig 5).

In the microalgal concentration experiment, no significant effects of pearl oyster preharvest conditions were observed on either the number of defects (F-test, p = 0.877), color (F-test, p = 0.623), darkness level (χ^2^ = 0.957) and luster (F-test, p = 0.297). (Fig 5A, 5B, 5C and 5D, respectively).

In the water temperature experiment, no significant effects of pearl oyster preharvest conditions were observed on either the color (F-test, p = 0.836) or darkness level (χ^2^ = 0.509) (Fig 5F and 5G, respectively). However, the number of defects and luster were significantly dependent on water temperature (F-test, p = 0.047 and p = 0.006, respectively) (Fig 5E and 5H, respectively). Furthermore, pearl oysters maintained at 22°C gave rise to a significantly lower proportion of pearls in the category with >10 defects and a significantly higher proportion in the category 6–10 defects, whereas pearl oysters maintained at 30°C produced a significantly lower proportion in the category 6–10 defects than expected. Finally, pearl oysters maintained at 22°C produced a significantly lower proportion of pearls without luster and a significantly higher proportion of pearls with high luster than expected by chance. Pearl oysters subjected to 30°C produced a significantly higher proportion of pearls without luster and a significantly lower proportion of pearls with high luster than expected by chance.

## Discussion

This study is the first analysis of the influence of food level (microalgal concentration) and water temperature, under controlled conditions, during the preharvest phase of pearl formation. We measured the expression levels of biomineralization-related genes in the pearl sacs, shell and pearl deposition rates, pearl nacre ultrastructure and quality traits. Our results show that, among the preharvest experimental factors investigated, water temperature had the greatest impact on pearl ultrastructure and quality. Indeed, pearl oysters maintained at 22°C during the last stages of pearl formation produced pearls with thinner nacre tablets, higher quality of luster and fewer defects than expected. However, pearl growth was higher for pearl oysters maintained at 30°C. Food level was shown to modulate the expression levels of the Aspein gene, with a significantly lower transcript abundance for pearl oysters fed at 28,000 cell mL^-1^ than those fed at 7000 cell mL^-1^. Nevertheless, we did not observe differences in pearl ultrastructure and quality between the two microalgal concentrations.

### Pearl sac biomineralization-related gene expression levels

As the donor oyster is the main genetic contributor to the expression of biomineralization-related genes involved in pearl formation [6], pearl oysters with grafts collected from each donor oyster were split across the two treatments for each experiment. Among the tested genes, only *Pmarg-Aspein* expression in the pearl sacs decreased when pearl oysters were fed abundantly (i.e., 28,000 cell mL^-1^ *T*. *lutea/C*. *gracilis*). This gene is involved in the calcite precipitation process [15] and is responsible for the formation of calcite in the prismatic shell layer [17]. This downregulation may indicate less calcite production and this could be advantageous for pearl production, as calcite is not desirable on the pearl surface and leads to a drop in quality. It would therefore be useful to measure other gene expression levels related to the formation of calcite. Food level was shown to significantly change the expression levels of several biomineralization-related genes in the mantle of *P*. *margaritifera* after a two-month exposure; for example, *Pmarg-MSI60* and *Pmarg-Pif177* showed a significant increase and decrease, respectively, for pearl oysters fed at 15,000 cell mL^-1^ in comparison to pearl oysters fed at 800 cell mL^-1^ [23]. In contrast, we did not provide evidence for such a food level effect on these same genes in the pearl sacs. Nevertheless, the microalgal concentrations we tested were not the same (7000 and 28,000 cell mL^-1^) and our experiment was shorter (one month).

In this study, the water temperature did not impact biomineralization gene expression in the pearl sac. This is in agreement with previous studies on the same five genes in the mantles of *P*. *margaritifera* subjected to 22 and 30°C [39,41]. However, in a previous study [41], a downregulation of *Pmarg-Aspein* and *Pmarg-Nacrein A1* was reported at 30°C in comparison to 26°C. Thus, it would be of interest to expand the range of tested water temperatures on the pearl sac biomineralization gene expression levels. Finally, with the advent of high-throughput sequencing technologies, an overall analysis without *a priori* knowledge, such as RNA-Seq, would be useful to identify differentially expressed genes related to biomineralization in response to food level and water temperature conditions.

### Shell and pearl deposition rate

Food level has been reported to strongly influence shell growth rate of several mollusk species [42–44]. In pearl oysters *P*.*margaritifera* and *P*.*maxima* fed with T-Iso from 10 to 100 × 10^3^ cell.mL^-1^, optimal food concentrations for maximum scope for growth were 10 to 20 × 10^3^ cell.mL^-1^ and 20 to 30 × 10^3^ cell.mL^-1^ respectively [45]. Moreover, it was shown that different microalgal concentrations between 800 and 15,000 cell.mL^-1^ (composed of a mix of T-Iso and *Chaetoceros gracilis*) influences shell growth, molecular mechanisms underlying shell biomineralization and nacre tablet thickness in the shell of *P*. *margaritifera* [23,24].

In this study, we did not find differences of shell and pearl growth rates between our two microalgal concentrations. The shell deposition rate was lower than in two other studies, despite higher microalgae concentration [23,24]. This could be explained by a difference in growth potential related to the difference in sizes of individuals in the three experiments (mean shell height: 85 ± 6 mm in [23]; 85 ± 5.7 mm in [24]; 121 ± 11 mm for experiment 1 and 120 ± 12 mm for experiment 2 in this study). Indeed, the daily rate of nacre deposition declined with the size of pearl oyster *P*. *margaritifera* [46]. Moreover, in young pearl oysters *P*. *margaritifera* (< two years old), the reproductive effort is low and almost all the energy is invested in somatic and shell tissue [47]. Shell deposition rate was higher for 23-month-old pearl oysters in comparison to 40 month old pearl oysters, with values of 12.2 μm day^-1^ and 2.15 μm day^-1^, respectively [48]. In this study, however, pearl oysters were approximately three years old and may have invested energy in both reproduction and shell growth. We did not report differences in pearl deposition rate between the two microalgal concentrations. We measured a pearl deposition rate of 0.78 ± 0.58 μm day^-1^ for pearls oysters fed at 7000 cell mL^-1^ and of 0.98 ± 0.51 μm day^-1^ for pearl oysters fed at 28,000 cell mL^-1^. These deposition rates for 12 month old pearls were in the same range as a previous study that found pearl deposition rates of 9.21 × 10^−2^ ± 0.01 and 1.44 ± 0.04 μm day^-1^ for 24-month-old and 4-month-old pearls, respectively, after a two-month exposure at 10,000 cell mL^-1^ [24].

The microalgal concentrations of this study did not affect pearl nacre tablet thickness or the number of nacre layers deposited per day. Conversely, trophic level was shown to influence nacre tablet thickness in the shell with thicker nacre tablets for pearl oysters fed at 15,000 cell mL^-1^ than the pearl oysters fed at 800 cell mL^-1^ after a two-month exposure [24]. The authors of the study concluded that higher shell deposition measured at 15,000 cell mL^-1^ seemed to be due to the combination of thicker aragonite tablets and an increasing shell deposition rate [24]. Testing a wider range of microalgal concentrations that was used in the present study would help to better understand the effect of food level on pearl biomineralization.

Water temperature is a key parameter for bivalve shell growth [49–52]. In this study, we reported a higher SDR for pearl oysters subjected to 30°C than those subjected to 22°C, with a gain of around 75%. Such an increase in shell growth with increased water temperature was also shown in the pearl oyster *P*. *margaritifera* with an increase by a factor of three between 21°C and 28°C [23] and by a factor of 2.9 between 22°C and 30°C [39]. Bivalve metabolic activity increases with an increase of water temperature until it reaches an optimum, beyond which it decreases [53,54]. The thermal optimum range was studied for several pearl oyster species [45,55]. For *P*. *margaritifera*, an energy gain from 22 to 30°C was reported, with metabolic rates maximized at 30°C [25]. This energy gain would explain the shell growth differences. Water temperature also influenced pearl growth rates, which was significantly higher for pearl oysters subjected to 30°C than to 22°C, with values of 1.38 ± 1.1 and 0.51 ± 0.4 μm day^-1^, respectively. These results confirm the influence of water temperature that was suspected to partially explain size differences in pearl nacre thickness and weight produced in locations with contrasted water temperatures [26].

In our study, water temperature significantly affected both pearl deposition rate and the number of tablets deposited per day, which were higher for pearl oysters subjected to 30°C than pearl oysters subjected to 22°C. These results are consistent with previous studies, which have shown that high water temperatures promote pearl nacre growth [30,56]. Moreover, a recent study has provided support for the assertion that water temperature influences pearl nacre tablet thickness [30]. In their study, the authors reported that the thickness of the nacre tablet layer was influenced by cold water, which induced thinner tablets [30]. This is in accordance with another study, which stated that low water temperature results in thinner laminar nacreous layers and that high water temperature led to faster pearl oyster growth and a higher rate of nacre deposition [57]. Finally, a strong correlation was found between nacre crystal misorientations and water temperature in a study including several mollusk species from different environments [58]. The strong correlation was only found with maximum water temperature, which suggests that higher water temperatures increase nacre deposition and that aragonite tablets grow faster [58].

For both experiments, pearl oyster growth and subsequent pearl deposition rates were positively correlated, which confirmed previous results from studies demonstrating positive correlations between pearl parameters (size, thickness or weight) and recipient oyster biometric parameters [26,39,59,60]. The energy allocated to pearl deposition rate is likely to depend on recipient oyster metabolism.

A strong positive correlation was also found between *Pmarg-MSI60* expression levels and PDR, and a negative correlation was found between *Pmarg-Aspein* expression levels and PDR. MSI60 is involved in the formation of the nacreous layer and includes a calcium-binding domain [13], whereas Aspein is related to the formation of the prismatic layer and contains an aspartic-rich domain, which might be involved in controlling the selective precipitation of calcite [15–17]. MSI60 could therefore be a potential molecular marker of the pearl nacre deposition rate.

### Pearl quality traits

The surface defects were not dependent on food level but on water temperature. Pearl oysters maintained at 22°C gave rise to a low proportion of pearls in the category of >10 defects, whereas pearl oysters maintained at 30°C produced a low proportion in the category of 6–10 defects. Rapid growth may result in pearls with a beaten surface appearance, referred to as “hammering” [19]. The slower growth rate of pearls at low water temperature may thus have resulted in fewer defects on the surface of the pearl. However, care should be taken when considering surface defects since some of them find their origin in the first stages of pearl formation [3].

The visual color and the darkness were not dependent on either food or water temperature. Pearl color has been reported to depend on donor oyster [32], on family effect [61] and is reported to be influenced by the environmental conditions where the recipient oysters grow [4,28]. The strength of the iridescent color has been shown to depend on the last layers of nacre and on its nacre structure in the shell [31]. The nacre structure was influenced by water temperature, with thinner nacre layers for pearl oysters reared at 22°C than at 30°C. However, there was no dependence of pearl color and water temperature. The darkness level has been found to be correlated with pearl nacre thickness and weight, with the palest pearls being the smallest [21,32]. These two pearl quality traits may be more dependent on the whole process of pearl mineralization rather than on the last stages of pearl formation.

Luster was dependent on water temperature but not food level. At 22°C, pearl oysters produced more pearls with high luster. Thinner nacre layers on the top of the pearl was suspected to enhance the luster [28], and several studies have reported that pearls are preferentially harvested when water temperature is low because of their better luster [19,27]. Moreover, the quality of half-pearls (mabe) produced from *Pteria penguin* was the best during a period of relatively low water temperature [62]. We verified this hypothesis experimentally and confirmed that water temperature influences both pearl nacre ultrastructure and luster, with thinner nacre tablets and better luster at low water temperatures. In French Polynesia, this could partially explain that pearls produced in the Gambier archipelago, where water temperature is lower than in the Tuamotu archipelago, are renowned for their high quality of luster.

## Conclusion

The last stage of pearl mineralization is crucial to obtain high-quality pearls and may be influenced by environmental parameters. Our results show that water temperature influences both pearl and shell deposition rates, with higher growth rates at higher water temperatures. Moreover, we confirmed that lower water temperature induces thinner nacre tablets, and we show that low water temperature gives rise to better luster and fewer defects. Nevertheless, we found no significant differences between the two tested microalgal concentrations on shell growth, pearl growth, nacre ultrastructure and quality traits. These results could have major implications for the pearl industry. A wise strategy to increase pearl quality would be to rear pearl oysters at high water temperatures to increase pearl growth and consequently pearl size; and to harvest pearls after a period of low water temperature to enhance luster and to reduce the number of defects.

## Supporting information

S1 Data Supporting_information.xlsx. (XLSX)
